# Dose-Dependent Progression of Chorioretinal Atrophy at the Injection Site After Subretinal Injection of rAAV2/8 in Nonhuman Primates

**DOI:** 10.1016/j.xops.2024.100516

**Published:** 2024-03-15

**Authors:** Immanuel P. Seitz, Fabian Wozar, G. Alex Ochakovski, Felix F. Reichel, Faik Gelisken, K. Ulrich Bartz-Schmidt, Tobias Peters, M. Dominik Fischer

**Affiliations:** 1 University Eye Hospital Tübingen, Centre for Ophthalmology, University of Tübingen, Tübingen, Germany; 2 Oxford Eye Hospital, Oxford University Hospitals NHS Foundation Trust, Oxford, UK; 3 Nuffield Department of Clinical Neurosciences, University of Oxford, Oxford, UK

**Keywords:** AAV, atrophy, gene therapy, inflammation, nonhuman primates

## Abstract

**Objective:**

Progressive retinal atrophy has been described after subretinal gene therapy utilizing the adeno-associated virus (AAV) vector platform. To elucidate whether this atrophy is a consequence of inherent properties of AAV, or if it is related to the surgical trauma of subretinal delivery, we analyzed data from an Investigational New Drug–enabling study for PDE6A gene therapy in nonhuman primates.

**Design:**

Animal study (nonhuman primates), retrospective data analysis.

**Subjects:**

Forty eyes of 30 healthy nonhuman primates (macaca fascicularis) were included in the analysis. Two AAV dose levels (low: 1x10E11, high: 1x10E12) were compared with sham injection (balanced saline solution; BSS). Twenty untreated eyes were not analyzed.

**Methods:**

Animals were treated with a sutureless 23G vitrectomy and single subretinal injections of AAV.PDE6A and/or BSS. The follow-up period was 12 weeks. Atrophy development was followed using fundus autofluorescence (AF), OCT, fluorescence angiography, and indocyanine green angiography.

**Main Outcome Measures:**

Area [mm^2^] of retinal pigment epithelium atrophy on AF. Presence of outer retinal atrophy on optical coherence tomography. Area [mm^2^] of hyperfluorescence in fluorescence angiography and hypofluorescence in indocyanine green angiography.

**Results:**

Progressive atrophy at the injection site developed in 54% of high-dose-treated, 27% of low-dose-treated, and 0% of sham-treated eyes. At the end of observation, the mean ± SD area of atrophy in AF was 1.19 ± 1.75 mm^2^, 0.25 ± 0.50 mm^2^, and 0.0 ± 0.0 mm^2^, respectively (sham × high dose: *P* = 0.01). Atrophic lesions in AF (*P* = 0.01) and fluorescence angiography (*P* = 0.02) were significantly larger in high-dose-treated eyes, compared with sham-treated eyes. Rate of progression in high-dose-treated eyes was 4.1× higher compared with low-dose-treated eyes.

**Conclusion:**

Subretinal injection of AAV.PDE6A induced dose-dependent, progressive retinal atrophy at the site of injection. Findings from multimodal imaging were in line with focal, transient inflammation within the retina and choroid and secondary atrophy. Atrophic changes after gene therapy with AAV-based vector systems are not primarily due to surgical trauma and increase with the dose given.

**Financial Disclosure(s):**

Proprietary or commercial disclosure may be found in the Footnotes and Disclosures at the end of this article.

We have previously reported on retinal atrophy developing after subretinal application of voretigene neparvovec (VN) in the post-approval setting.^1^ This retinal atrophy seems to be progressive in nature, affects a relevant subset of patients, and in early stages, is often locally associated with the injection site.^2^^,^^3^ Although thus far, visual acuity and gains in dim light sensitivity have been maintained, the long-term growth potential of this atrophy is not yet clear. It could pose an undesirable side effect and potentially impact on therapeutic efficacy. A multitude of hypotheses regarding its cause have been discussed: Is it a direct result of the use of an adeno-associated virus (AAV) vector system in general,^4^ such as direct toxicity or an immune response?^5^, ^6^, ^7^, ^8^ Or is it primarily caused by the surgical trauma associated with the penetration and forced separation of the delicate neuroretina from the retinal pigment epithelium and the stretching associated with bleb formation? Both might hamper progress in a host of other indications, where AAV-based vectors are applied by subretinal injection. As such, the answer is highly relevant to the field looking to minimize adverse effects and maximize the benefit for our patients.

To further elucidate the mechanism of this progressive atrophy, we reviewed previous Investigational New Drug–enabling studies performed in healthy nonhuman primates (NHP). In these experiments, clinical grade rAAV2/8 designed to treat PDE6A-associated retinitis pigmentosa was administered subretinally as a single dose of either 1x10E11 (low dose) or 1x10E12 (high dose) vector genomes (vg). In addition, sham treatment (subretinal balanced salt solution) was performed in eyes contralateral to AAV-treated eyes to investigate surgical trauma as a potential confounding factor. The development of atrophic changes was monitored for 12 weeks and quantified using fundus autofluorescence (AF) imaging. Presumed atrophic lesions in AF were verified using OCT. To complement AF, fluorescein angiography (FA) and indocyanine green angiography (ICGA) were performed and evaluated as well.

We hypothesized that if the development of progressive atrophy was primarily due to the surgical trauma, all 3 groups would demonstrate such atrophy. However, if the vector system was the main reason for the atrophic changes, only the AAV-treated animals would demonstrate atrophy.

## Methods

A total of 30 animals were treated with single subretinal injections and observed over a 12-week period. Two AAV dose levels (low dose: 1x10E11, and high dose: 1x10E12 vector genomes [vg]) were compared with sham-injected (balanced saline solution) controls. In AAV-treated eyes, small balanced salt solution preblebs were raised to access the subretinal space and minimize inadvertent injection of vector in the vitreous cavity during bleb formation. In animals that received AAV injections in one eye, the contralateral eye was either left untreated (results not reported) or used for sham injections. Only animals for which the full course of observation could be completed were included in this analysis. In total, 18 eyes were treated with sham injections, 11 with the low dose and another 11 eyes with the high dose. All AAV-treated eyes were left eyes; for sham eyes, 6 were left and 12 were right eyes. The same disposable supplies were used for all injections. The total volume injected was 170 μL per eye, which was achieved in all animals, with no relevant reflux noted by the surgeon. All animals received steroids. Two milligrams of dexamethasone subconjunctival were applied at the end of the subretinal injection procedure. In addition, animals received 1% prednisolone eye drops 3× daily for 1 week, and transient systemic immunosuppression with prednisone 1 mg/kg (intramuscular) from day 2 to day 5.

**Figure 1:**
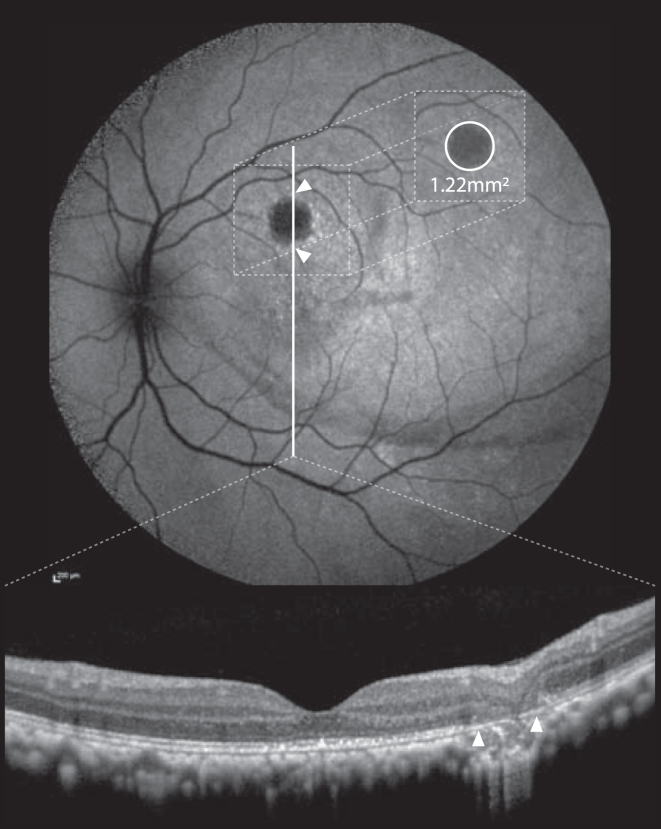
Atrophy at the injection site was quantified using fundus autofluorescence imaging (AF, top) and verified using OCT (bottom). Measurements were taken with the proprietary Heidelberg Engineering Eye Explorer (HEYEX 2). Sample measurement: 1.22 mm^2^. Areas of atrophy were verified using OCT (white arrows) to exclude false positive calls of atrophy in areas with confounding effects interfering with AF signal (e.g., pigment displacement – AF image: faint hypofluorescent arch along inferior bleb margin).

Fundus autofluorescence images were taken at baseline, week 2, week 4, week 8, and week 12 (end of observation). The area of retinal pigment epithelium (RPE) atrophy was measured in the proprietary Heidelberg Engineering Suite. Fundus autofluorescence is the standard modality to quantify the area of retinal atrophy in VN patients. To exclude other causes of AF hypofluorescence (false positives), putative atrophic lesions were cross-referenced with corresponding OCT images for anatomic verification (Fig 1). In the same manner, hyperfluorescent lesion size was measured in late-phase FA and hypofluorescent lesion size in indocyanine green angiography. The former served as a surrogate for either leakage (i.e., inflammatory disruption of diffusion barrier) or window defects (following atrophy of the retinal pigment epithelium). The latter served as a surrogate for choroidal hypoperfusion due to trauma (early) or inflammation and/or chorioretinal atrophy (later).

The studies were conducted at the Labcorp Early Development Services GmbH facility in Münster, Germany, in adherence to directive 2010/63/EU for the protection of animals used for scientific purposes, and the 2007/526/EC Commission Recommendation (Appendix A of Convention ETS 123). The studies were in compliance with the German Animal Welfare Act and approved by the local Institutional Animal Care & Use Committee (LANUV).

Statistical analysis was performed using Graphpad Prism 10.

## Results

### Fundus Autofluorescence

Atrophy at the injection site was quantified using AF. Adeno-associated virus–treated eyes of both low and high dose developed retinal atrophy at the injection site. In contrast, none of the sham-treated eyes displayed significant atrophy at the injection site. Furthermore, high-dose (1x10E12)–treated eyes displayed a higher incidence and larger areas of retinal atrophy compared with low-dose (1x10E11)–treated and sham-treated eyes. Table 1 summarizes the cohort differences at the end of observation (week 12).

**Table 1:** Share of Eyes Affected by Injection Site Atrophies [%], Atrophic Area at Week 12 [mean ± SD, mm^2^], and One-Way ANOVA With Multiple Comparisons at Week 12

Group	Share of Eyes with Injection Site Atrophy		Atrophic Area at Week 12 [mean ± SD, mm^2^]
Sham	0/17	0%	0 ± 0
Low dose	3/11	27.3%	0.25 ± 0.50
High dose	6/11	54.5%	1.19 ± 1.75

One-Way ANOVA of Atrophy Area, Multiple Comparisons, Week 12	
Sham vs. high dose	*P* = 0.0085^a^
Sham vs. low dose	*P* = 0.7816
Low dose vs. high dose	*P* = 0.0792

ANOVA = analysis of variance.

Full-thickness atrophies were not detected at the injection site in sham-treated animals. This indicates that surgical trauma alone is insufficient to explain their development. Atrophy developed twice as often in high-dose (1x10E12)-treated compared with low-dose (1x10E11)-treated eyes, and atrophies were larger in high-dose-treated animals. This difference in atrophy area was statistically significant for sham- vs. high-dose-treated eyes.

a Indicates statistical significance.

**Figure 2:**
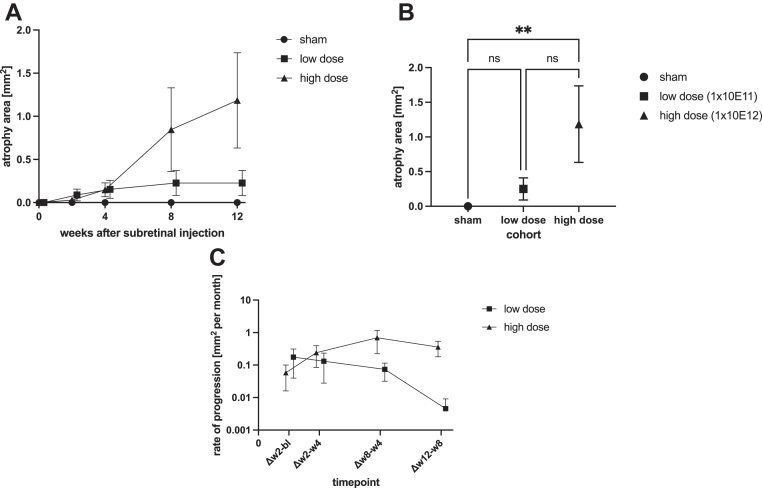
**A,** Injection site atrophy area as quantified by autofluorescence (AF) over time by group. Mean ± standard error of the mean (SEM). There was a dose-dependent development of atrophy at the injection site. Starting by week 8, high-dose-treated animals developed relevant atrophic lesions around the injection site. Low-dose-treated animals displayed small lesions sizes, with no significant progression. No atrophy developed at the injection site in sham-injected animals. **B,** One-way ANOVA with multiple comparisons of atrophic lesion size at the end of observation (week 12) by group. Mean ± SEM. At the end of observation there was statistically significant (*P* = 0.01) development of atrophy at the injection site in high-dose-treated animals, compared to sham-injected animals. This was not observed in low-dose-treated animals (*P* = 0.78). **C,** Rate of progression, log scale. Progression rate seemed to follow a linear function despite its quadratic nature (area). This indicates a slowdown of the progression rate in both treatment groups between weeks 8 and 12. ANOVA = analysis of variance.

Atrophy at the injection site usually became evident in week 2 or 4. It displayed only minimal progression in both high- and low-dose-treated eyes until week 4. Yet by week 8, there was a pronounced divergence in the rate of progression between eyes from both treatment groups. Although atrophy in low-dose-treated eyes seemed to stabilize over time, a subset of atrophic lesions in high-dose-treated eyes exhibited accelerated progression, which continued up to the end of observation (week 12). The progression of atrophic lesions is illustrated in Figure 2A. Consequently, by week 12, area of atrophy in high-dose-treated eyes had diverged from sham-treated eyes with statistical significance. The same was not observed for low-dose-treated eyes (Fig 2B). Despite area measurements scaling with the square of the diameter, atrophy rate of progression was best approximated using a linear fit, suggesting a slowdown of the progression rates in the treated groups between weeks 8 and 12 (Fig 2C). However, expressed linearly over 12 weeks, the rate of progression was estimated to be around 4.1× times higher in high-dose-treated vs. low-dose-treated eyes (0.38 mm^2^ per month vs. 0.09 mm^2^ per month, *P* = 0.01).

**Figure 3:**
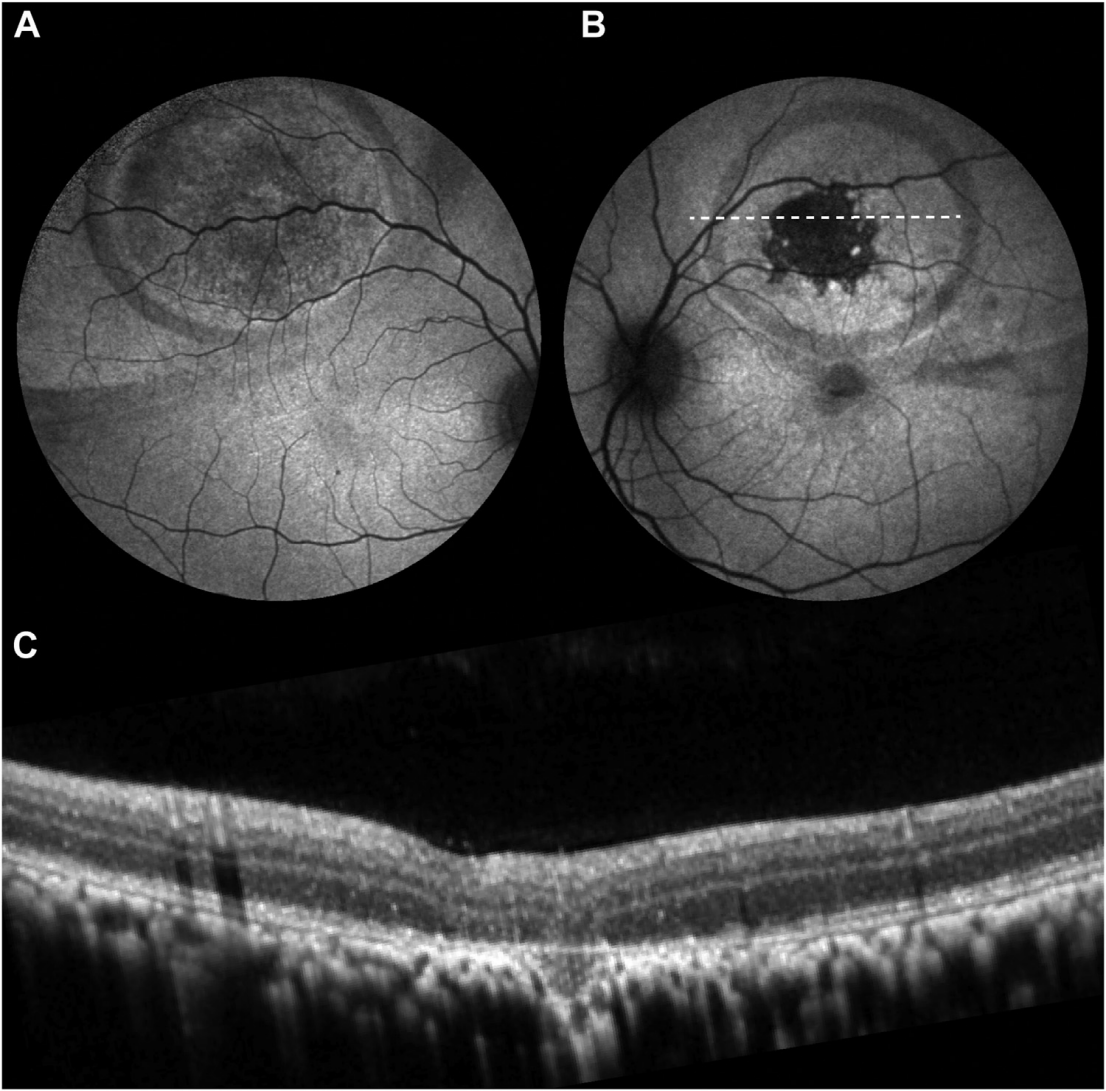
Sample difference in findings between sham- and high-dose-injected eyes of the same animal. **A,** Sham-treated eye: Surgery-related changes on fundus autofluorescence (AF) included mechanical displacement of pigment and were observed in all eyes, including sham. The displaced pigment settled mostly along the prebleb and bleb margins (2 hypofluorescent rings). **B,** High-dose-treated eye: In addition to pigment displacement, there is a clearly delineated lack of fluorescence indicating atrophy of the retinal pigment epithelium (RPE). Dotted line: location of OCT scan displayed in (C). **C,** Corresponding OCT image demonstrates that the area of RPE atrophy indicated by lack of AF signal also shows atrophy of the outer retina (ellipsoid zone, external limiting membrane, outer nuclear layer, and outer plexiform layer). Together this demonstrates retinal atrophy at the injection site (black area in the bleb center).

**Figure 4:**
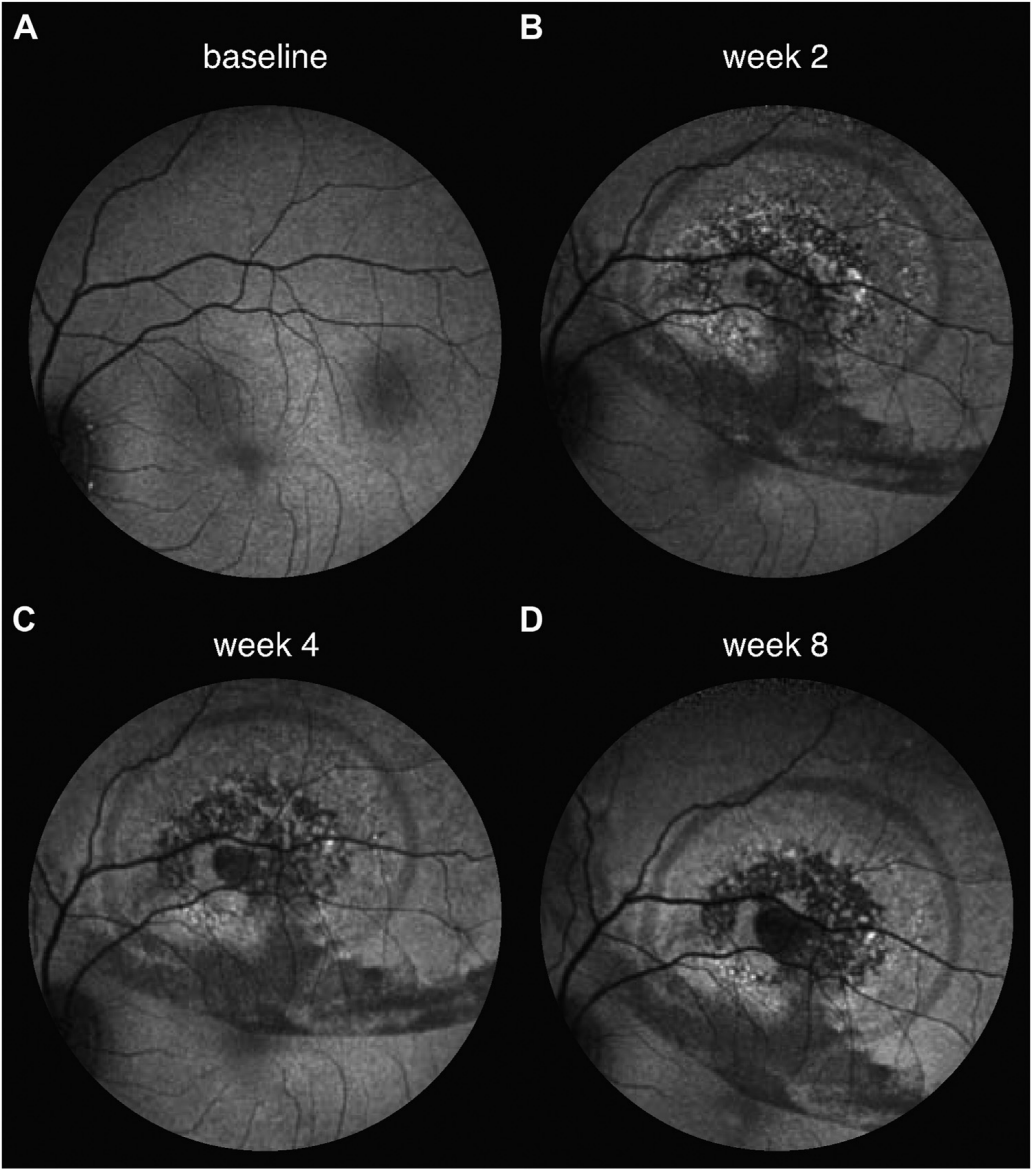
Mottled phenotype leading into atrophy. In one eye, enlargement of the atrophic area was preceded by pronounced “salt-and-pepper”-like mottling. The area of this lesion was captured on fundus-autofluorescence at baseline **(A)**, week 2 **(B)**, week 4 **(C)** and week 8 **(D)**. Both the central defect and the mottled changes surrounding it did slowly enlarge over the course of observation. Of note, this eye was the only one to exhibit this peculiar phenotype. In other eyes, the atrophy margins were unremarkable prior to degeneration.

Figure 3 provides an example of the difference between sham vs. high-dose treatment in fellow eyes of the same animal. Figure 4 illustrates the progression of atrophy in one AAV-treated eye. This eye was chosen for illustration because here atrophy was preceded by a peculiar “salt-and-pepper”–like mottling in its immediate vicinity. Eventually, the mottled area was revealed to be composed of microlesions that enlarged and coalesced over time. Of note, this was not observed in any other eye. In all other cases, the area surrounding the atrophy margin was unremarkable prior to degeneration.

With regards to surgical complications, no relevant reflux was noted by the surgeon. Small intraretinal hemorrhages at the retinotomy site were observed in a total of 6 treated eyes (5 sham, 1 low dose). None of these eyes developed atrophy. Although not directly observed at any time point, OCT findings that were in line with a resolved macular hole were seen in 3 sham eyes. None of the noted surgical complications predicted the development of atrophy. Also, the animals’ sex had no influence on atrophy development (*P* = 0.49).

### Fluorescein Angiography

**Figure 5:**
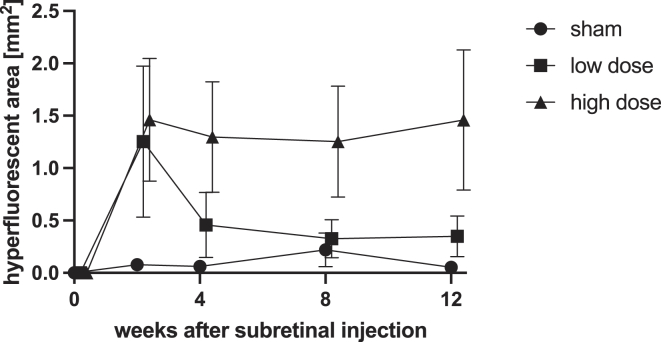
Area of hyperfluorescence during fluorescein angiography (FA) quantified over time by group. Mean ± standard error of the mean. Sham-treated animals displayed only minimal changes in FA. For adeno-associated virus–treated eyes, hyperfluorescence peaked at week 2. In low-dose-treated animals, hyperfluorescence had largely resolved spontaneously by week 4. In high-dose-treated animals, hyperfluorescent lesions persisted until week 12 (the end of observation).

Fundus autofluorescence revealed no gross hyperfluorescent lesions in sham-treated animals. In AAV-treated animals, hyperfluorescent lesions were most pronounced in week 2. In the early weeks of monitoring, areas of FA hyperfluorescence exceeded that measured by AF (e.g., compare area at week 2 between Figs 2 and 5). Over time, FA and AF lesion converged in size. By week 4, there was a statistically significant divergence between the low- and high-dose-treated groups: Although low-dose-treated eyes recovered almost completely, defects in high-dose-treated eyes remained unchanged and did not recover until the end of observation. Overall, both dose group (*P* = 0.02) and time point (*P* < 0.01) were confirmed to be significant factors influencing hyperfluorescent lesion size (2-way analysis of variance). Multiple comparisons with regards to the dose-dependency by time revealed significant differences between sham and high-dose lesion size at weeks 2 (*P* = 0.04) and 4 (*P* = 0.04), with slightly higher values for weeks 8 (*P* = 0.09) and 12 (*P* = 0.07).

### Indocyanine Green Angiography

Indocyanine green angiography revealed time-dependent, focal hypofluorescence at the injection site in all dose groups. Hypofluorescent areas were initially larger than atrophic areas measured by AF. Like in FA, this was most pronounced at week 2, followed by partial recovery. Unlike FA and AF, ICGA-hypofluorescence was also detected in sham-treated animals, and there was no divergence of findings between high- and low-dose-treated eyes. Descriptive analysis hinted at a weak dose-dependency, with dose group means at all time points obeying a dose-dependent hierarchy (high dose > low dose > sham). Yet this difference was not statistically significant.

## Discussion

Gene therapy has long been heralded as a next frontier in personalized medicine and has indeed provided means for successful treatment in orphan indications such as *RPE65*-associated inherited retinal degeneration. We and others have identified atrophic changes in patients following AAV-mediated retinal gene therapy.^1^, ^2^, ^3^ Dr Bainbridge was the first to describe atrophic changes after following up his patients.^9^ Specifically, his observations included features of gene therapy–associated uveitis such as transient anterior and/or posterior uveitis, vitritis, optic disc swelling, vascular sheathing—all responsive to steroid treatment. Importantly, he also described the gradual development of focal chorioretinal pigmentary changes in one patient.^9^

Subsequent studies by another team with a similar gene therapy product (also AAV serotype 2, also encoding for RPE65) successfully demonstrated efficacy with an acceptable safety profile. Voretigene neparvovec was approved by US Food and Drug Administration and European Medicines Agency and successfully applied to hundreds of patients worldwide. Although the pivotal studies mentioned listed adverse effects relating to the retina (incidence of 3/81 for macular hole, transient subretinal deposits, or epiretinal membrane; 2/81 for foveal atrophy; 1/81 eyes for foveal schisis or retinal haemorrhage), atrophic changes at the injection site, the area covered by the bleb and even areas beyond in a significant subset of patients have since been reported.

PERCEIVE is a Post-Authorization, Multicenter, Multinational, Longitudinal, Observational Safety Registry Study for Patients Treated with VN (EUPAS31153) that was mandated by the European Medicines Agency to collect long-term safety information associated with VN. Once these atrophic changes were reported, a term from the Medical Dictionary for Regulatory Activities had to be used to collect the data. The most appropriate (or least inappropriate) term within it was deemed to be “chorioretinal atrophy” (CRA). However, although several experts thought this term may be a misnomer as it was unclear whether the choroidal structures are at all affected, this broad term allowed for a lumping strategy: Several potentially different clinical findings would all be classified under one term (potential overreporting its frequency) compared with a splitting strategy (with potential underreporting of CRA by calling it, e.g., foveal atrophy in one patient and macular hole in a different patient). Recent reporting suggests that more than 10% of patients treated with VN present with CRA in the years following gene therapy. It remains unclear to date what causes these atrophic changes. This raises a pressing question for the wider field of AVV-based gene therapy: Is it unique to treatment with VN and the RPE65-deficent retina, or is it a consequence of inherent immunogenic or otherwise toxic properties of the AAV vector system and/or its preparation, and therefore potentially not limited to VN?^4^ Multiple hypotheses have been put forward to explain the occurrence of CRA after gene therapy with VN. It has been reported that the degree of dim-light sensitivity improvements correlates strongly with the development of CRA.^10^ The authors of this study suggest that CRA could be a toxic metabolic sequela of RPE65 (over-)expression. Other studies point toward inflammation as a potential driver of CRA after AAV-based gene therapy.^8^^,^^11^ This inflammation can either arise from an antiviral immune response or from immunogenic impurities such as extraviral DNA.^12^

To help elucidate the mechanism underlying the development of CRA, we analyzed the development of atrophic changes in healthy NHP retinae treated with a clinical grade AAV-based retinal gene therapy vector (AAV8.PDE6A). If atrophic changes were exclusive to gene therapy for *RPE65*-associated retinal disease, we would expect not to see them in healthy NHP using another AAV vector. Yet if progressive atrophy was observed after injection of another AAV-based gene therapy vector in an otherwise healthy non-RPE65 retina, this would suggest that inherent properties of the vector system are likely to play a key role in the development of progressive atrophic changes after gene therapy.

Some degree of atrophy at the injection (i.e., the “touchdown”) site may well be expected due to the surgical trauma associated with the subretinal injection itself.^3^^,^^13^ Increased injection pressures have been known to cause atrophy at the injection site in both monkeys^14^ and pigs.^15^^,^^16^ Therefore, many gene therapy treatment centers try to minimize injection pressures through the use of injection systems with pneumatic-assisted foot pedals.^17^ Although spontaneous enlargement of mechanical RPE defects is not unheard of (e.g., in myopes), this type of mechanical atrophy would be presumed to be mostly stationary or self-limiting^14^ rather than progressive and be present in both sham and AAV-treated groups. Curiously, this is not what we found. While previously described surgically induced changes such as displacement of pigment or minimal stationary lesions^14^ were present in eyes of all treatment groups (sham, low-dose AAV, high-dose AAV), only AAV-treated eyes developed significant atrophic areas (Fig 3). Furthermore, these lesions were dose-dependent (Fig 2B) both in terms of frequency and size. In addition, the temporal dynamics of atrophy development (Fig 2A) and (in some cases) morphological appearance (Fig 4) were reminiscent of cases presented with atrophic changes following VN gene therapy.^1^, ^2^, ^3^ Given sparse findings in sham-treated eyes, surgical trauma alone is insufficient as an explanation for their occurrence.

With regards to dose, there was a marked divergence in findings between low- and high-dose-treated eyes. In low-dose-treated eyes, lesions were generally small and progressing very slowly, seemingly reaching a plateau by week 8. Indeed, in the third month of observation, the rate of progression in low-dose-treated eyes had regressed toward 0 (Fig 2C). In high-dose-treated eyes, rate of progression was significantly higher (4.1× times, *P* = 0.01) compared with low-dose-treated eyes. Yet, comparing month 2 and 3 of observation, there was a slight decline in rate of progression in the high-dose-treated eyes, suggesting that atrophy enlargement may eventually slow down over time. The difference between high- and low-dose-treated eyes was confirmed in FA. Shortly after injection, there was pronounced hyperfluorescence in both AAV-treated groups, with no relevant leakage in sham-treated eyes (Fig 5). Initially, these areas of hyperfluorescence on FA were much larger, compared with areas of atrophy quantified by AF. This was interpreted as AAV-related focal inflammation and leakage. Months after (week 8 and 12), late hyperfluorescence became the dominant finding during FA, indicating window defects due to RPE atrophy. In line with this, quantification of areas with FA hyperfluorescence aligned with lesion size in AF toward the later time points (weeks 8 and 12). In low-dose-treated eyes, this meant that FA abnormalities recovered over time, whereas for high-dose-treated eyes, lesion size remained constantly high up to the end of observation.

**Figure 6:**
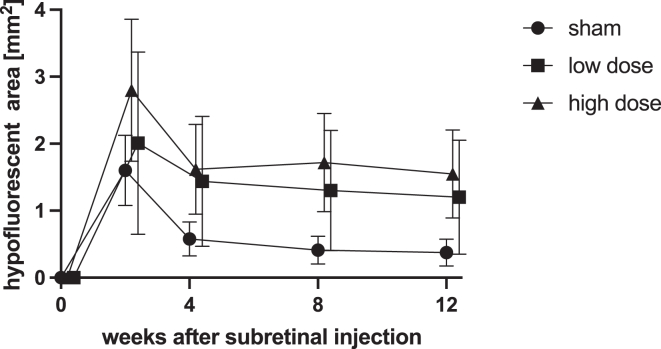
Choroidal hypofluorescence area as quantified by indocyanine green angiography (ICGA) over time by group. Mean ± standard error of the mean. Hypofluorescence in ICGA preceded detection of atrophy by fundus autofluorescence (AF). In contrast to AF and fluorescein angiography, ICGA hypofluorescence was also evident in sham animals. However, hypofluorescent appeared somewhat transient as lesion size peaked at week 2 followed by partial recovery. This time-dependency was statistically significant (2-way ANOVA: *P* = 0.00). Although differences between groups were not significant, there was a trend toward dose-dependent lesion size and persistence in hypofluorescence in adeno-associated virus–treated eyes. ANOVA = analysis of variance

Indocyanine green angiography was the only modality to suggest that surgery influenced outcomes. Focal ICGA hypofluorescence as a surrogate for choriocapillary hypoperfusion was seen in all groups, including sham-treated eyes. Here, differences between groups were not statistically significant. Yet descriptively, AAV-treatment seemed to initially intensify the surgically induced hypoperfusion (Fig 6). We speculate that this could point toward a combined etiology of short-term surgery-induced hypoperfusion exacerbated by dose-dependent focal chorioretinitis (inflammation). At later time points, ICGA provided clear evidence that atrophic lesions following AAV gene therapy affected both retina and choroid. This suggests that the Medical Dictionary for Regulatory Activities term “chorioretinal atrophy” may not be the misnomer we initially thought it was.

We see several limitations to this study. Healthy NHP eyes differ from eyes in patients suffering from an inherited retinal disease. It follows that the changes described in this study cannot claim to be a full reproduction of the clinical situation. Similarly, although the vector used in this study also is based on AAV, it differs from VN in a number of ways (e.g., capsid pseudotype 8 vs. 2, expression cassette with coding sequence for PD6A vs. RPE65). Differences in manufacturing of the viral vector can include other potentially confounding factors. Research grade AAV solutions were demonstrated to be potentially more immunogenic compared with clinical grade product.^12^ Although this study was performed with clinical grade AAV vector manufactured under strict GMP guidelines, even clinical grade preparations contain residual impurities (e.g., host cell protein, plasmid-derived DNA, host cell genomic DNA), which could be an avoidable source of inflammation and atrophy.^12^ However, these limitations do not preclude the finding of AAV-associated dose-dependent chorioretinal atrophy after subretinal gene therapy using clinical grade AAV vector. The changes could not be explained by the surgery alone and were progressive in nature.

In summary, our results suggest that inherent properties of the AAV vector platform can likely cause or aggravate the development of progressive chorioretinal atrophy. The fact that this is not observed in the sham group rules out surgical trauma alone as its main cause. The dose-dependent nature further highlights a causal relationship with AAV and/or its preparation. The fact that this was observed with a clinical grade AAV of a different pseudotype (AAV8) to that used for VN (AAV2) strengthens the notion that the issue of CRA may not be limited to VN.
